# New Pesticides, Old Problems: Despite Warnings, Use during Pregnancy Persists

**Published:** 2008-12

**Authors:** Bob Weinhold

The sight of scurrying cockroaches trumps warnings against using pesticides during pregnancy. That’s one insight from a study of pesticide use before and after the U.S. Environmental Protection Agency’s 2001 and 2002 retail sales restrictions of chlorpyrifos and diazinon **[*EHP*** **116:1681–1688; Williams et al.]**. The team of U.S. researchers also found that use of replacement pesticides is steadily increasing to fill the void, that the air the test subjects breathed remains surprisingly contaminated with chlorpyrifos and diazinon up to 5 years after the restrictions went into effect, and that household use of pesticide spray cans and bug bombs contaminated the air far more than did use of bait traps or boric acid or spraying by professional exterminators.

In the study, 511 pregnant inner-city women wore personal air samplers for a 48-hour period during their third trimester and reported pesticide use and sightings of pests throughout pregnancy. The researchers compared their findings from subjects enrolled in the study between 2000 and 2001 with those from subjects enrolled between 2002 and 2006. This reflected the timing of the pesticide bans—retail sales of chlorpyrifos were phased out at the end of 2001 and diazinon at the end of 2002. Participants received regular newsletters containing pertinent information, including warnings about potential health effects of residential pesticide use and information on alternative pest control methods.

For 6 months after chlorpyrifos went off the market, use of replacement pesticides fell, perhaps due to elevated awareness of pesticide dangers. But a steady, significant increase in sightings of cockroaches, the most commonly observed pest, was correlated with a steady, significant increase in the use of replacement pesticides for every 6-month period from 2002 through 2006. Throughout the study period, at least 85% of the women reported using pesticides.

Chlorpyrifos and diazinon were found in more than 98% of air samples both before and after the phase-outs, and 18–75% of the personal air samples contained at least 1 replacement pesticide. The chemicals the researchers measured included permethrin (a commonly used pyrethroid) and piperonyl butoxide (a pyrethroid synergist, or chemical added to a pesticide to increase its effectiveness). The authors say this is the first study to document extensive residential exposure to piperonyl butoxide.

Data on health effects of these pyrethroid products at the measured concentrations are limited. However, there is growing evidence of health and environmental damage from these products, which are proving to be ubiquitous both indoors and out. The authors say their findings indicate that these products warrant additional research on their use, occurrence, and health effects. They also noted that pest resistance to pyrethroids may be playing a role in pest increases.

## Figures and Tables

**Figure:**
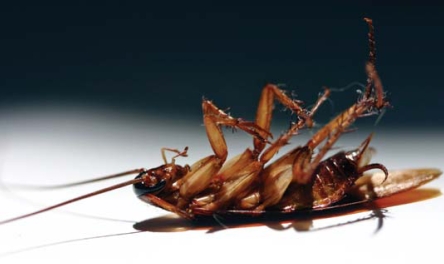
This and other studies have reported a strong association between the degree of housing disrepair and both pest sightings and use of pest control measures.

